# Transcriptome-Wide Identification and Prediction of miRNAs and Their Targets in *Paris polyphylla* var. *yunnanensis* by High-Throughput Sequencing Analysis

**DOI:** 10.3390/ijms18010219

**Published:** 2017-01-22

**Authors:** Li-Zhen Ling, Shu-Dong Zhang, Fan Zhao, Jin-Long Yang, Wen-Hui Song, Shen-Min Guan, Xin-Shu Li, Zhuang-Jia Huang, Le Cheng

**Affiliations:** 1 BGI-Yunnan, Kunming 650106, China; zhaofan@genomics.cn (F.Z.); Yangjinlong2@genomics.cn (J.-L.Y.); songwenhui@genomics.cn (W.-H.S.); guanshenmin@genomics.cn (S.-M.G.); lixinshu@genomics.cn (X.-S.L.); huangzhuangjia@genomics.cn (Z.-J.H.); 2 China National GeneBank, BGI-Shenzhen, Shenzhen 518083, China; 3 Germplasm Bank of Wild Species, Kunming Institute of Botany, Chinese Academy of Sciences, Kunming 650201, China; sdchang@mail.kib.ac.cn; 4 College of Clinical Medicine, College of Basic Medical Sciences, Dali University, Dali 671000, China

**Keywords:** *Paris polyphylla* var. *yunnanensis*, miRNAs, seed dormancy, high-throughput sequencing technology

## Abstract

Long dormancy period of seeds limits the large-scale artificial cultivation of the scarce *Paris polyphylla* var. *yunnanensis*, an important traditional Chinese medicine. Characterizing miRNAs and their targets is crucial to understanding the role of miRNAs during seed dormancy in this species. Considering the limited genome information of this species, we first sequenced and assembled the transcriptome data of dormant seeds and their seed coats as the reference genome. A total of 146,671 unigenes with an average length of 923 bp were identified and showed functional diversity based on different annotation methods. Two small RNA libraries from respective seeds and seed coats were sequenced and the combining data indicates that 263 conserved miRNAs belonging to at least 83 families and 768 novel miRNAs in 1174 transcripts were found. The annotations of the predicted putative targets of miRNAs suggest that these miRNAs were mainly involved in the cell, metabolism and genetic information processing by direct and indirect regulation patterns in dormant seeds of *P. polyphylla* var. *yunnanensis*. Therefore, we provide the first known miRNA profiles and their targets, which will assist with further study of the molecular mechanism of seed dormancy in *P. polyphylla* var. *yunnanensis*.

## 1. Introduction

MicroRNAs (miRNAs) are a class of regulators referred to as “dimmer switches” of gene expression. They are short (about 18–27 nt), noncoding and endogenous RNA molecules. miRNAs are initially transcribed as much longer single-stranded RNAs with imperfect hairpin structure, from which the mature miRNAs are excised by Dicer-like enzymes in plants [1]. They target protein-coding genes with Watson–Crick complementary bases to their messenger RNAs for the posttranscriptional repression. In plants, miRNAs bind to their target mRNAs with a high degree of complementarity, and several computational approaches have been developed to predict target sites throughout the genome [2,3]. It was estimated that at least 1% of protein-coding genes are regulated by miRNAs in plants [4,5]. In particular, many transcription factors (TF), such as myeloblastosis-related proteins (MYB), growth-regulating factor (GRF), squamosa promoter binding protein-box (SBP-box), Apetala2 (AP2), and MADS-box gene families, have been shown to be regulated by miRNAs in plants [6]. Accumulating evidence has indicated that miRNAs play an important role in gene regulation in the development of many plant tissues and organs [1,7]. A recent study has reported that the conserved miR156 and miR172 families have been characterized functionally during seed dormancy. Overexpression of MIR156 genes promoted seed dormancy, whereas overexpression of MIR172 delayed seed dormancy [8].

*Paris polyphylla* var. *yunnanensis*, also named “Chonglou”, is a Chinese traditional medicinal herb. It is a perennial herbaceous plant and belongs to the Melanthiaceae family, easily growing in moist woodland and widely distributed in southwestern China [9]. It has been reported that the rhizome of *Paris polyphylla* var. *yunnanensis* contains active pharmaceutical ingredients utilized to activate blood circulation, remove dispersing blood stasis and hemostasis, relieve pain, and treat liver and lung cancer. As the main source of bioactive compounds, the wild plants have been endangered because of overcollection in the past decades. To guarantee the sustainable use of natural resources of *P. polyphylla* var. *yunnanensis*, the successful cultivation of this plant is imperative. However, only 40% of seeds can germinate after experiencing dormancy for 18 months or even longer than two years in a natural environment [10]. Studies have indicated that the seed of *P. polyphylla* var. *yunnanensis* is of the typical morphophysiological dormancy (MPD). Freshly-harvested seeds of *P. polyphylla* var. *yunnanensis* are embraced by the mesophyll and bright red outer layer coats and contain a large endosperm and an undeveloped globular embryo [11,12]. These seeds need to complete the morphological after-ripening process in the first winter and can develop into the hypocotyl, radicle, and cotyledon [10,13]. Then the seeds will not immediately germinate until the next winter, when the physiological after-ripening process has been basically completed [14]. A previous study revealed that the germination of the seeds was significantly delayed when seed coats had not been removed in suitable germination conditions [11]. In addition, the endogenous inhibitors present in the mesophyll outer layer coat can inhibit the seed germination of *P. polyphylla* var. *yunnanensis* [11,15]. The studies have suggested that organic acid, alkaloid, and phenolic compounds are the main germination inhibitors, of which alkaloid exhibits the strongest inhibition effect on the growth of the radical and hypocotyl, and seed germination [15]. In addition, the germination inhibitors were also found in the seeds [11,12]. Although the seeds and seed coats contain germination inhibitors, they showed different germination inhibitory effects. Therefore, a comparison of miRNA-mediated developmental regulation between seeds and seed coats might be useful to explore the functions of miRNAs in seed dormancy in *P. polyphylla* var. *yunnanensis*.

According to the Plant DNA C-values database [16], *P. polyphylla* has a big genome size (more than 60 GB) [17]. In addition, *P. polyphylla* var. *yunnanensis* is not a model plant and most work has focused on the identification and isolation of the bioactive compounds due to its medical value [18,19,20]. At present, cost-effective high-throughput sequencing technologies provide a means to explore the functional genes by sequencing the transcriptome of an organism without a reference genome. Many important functional genes related to seed dormancy release have been mined via RNA-Seq sequencing [21]. Therefore, characterizing the miRNAs and their target genes in dormant seeds and their seed coats is necessary to gain a better understanding of the functional roles of miRNAs during seed dormancy. In the present study, we sequenced the small RNA and transcriptome data from both dormant seeds and their seed coats using high-throughput RNA-Seq technologies. Based on the assembled transcripts, we characterized conserved and novel miRNAs in seeds and seed coats of *P. polyphylla* var. *yunnanensis* by bioinformatics tools. Meanwhile, the corresponding miRNA targets were identified and annotated in this study. Therefore, this study will provide a basis to understand the functions of miRNAs in seed dormancy in *P. polyphylla* var. *yunnanensis*.

## 2. Results

### 2.1. Transcriptome Sequencing, Assembly, and Annotation

Seeds and their corresponding seed coats of *P. polyphylla* var. *yunnanensis* were subjected to deep sequencing of messenger RNA (RNA-Seq), using three biological replicates. In each mRNA library, a total of 63.33 M raw reads were obtained and the filtered clean reads (about 59 million) with 97% of Q 20 were used (Table S1). After combining the three replicates and removing redundancy, 146,671 unigenes with an average length of 923 bp (90,419 from seeds and 135,008 from seed coats) were yielded (Table 1). In this study, more than 50% of unigenes were annotated in the Nr database and showed significantly similarity to known proteins of various plants including *Phoenix dactylifera* (45.83%) and *Musa acuminata* subsp. *Malaccensis* (13.57%), *Nelumbo nucifera* (4.71%), and *Vitis vinifera* (4.68%) (Figure S1).

**Figure 1:**
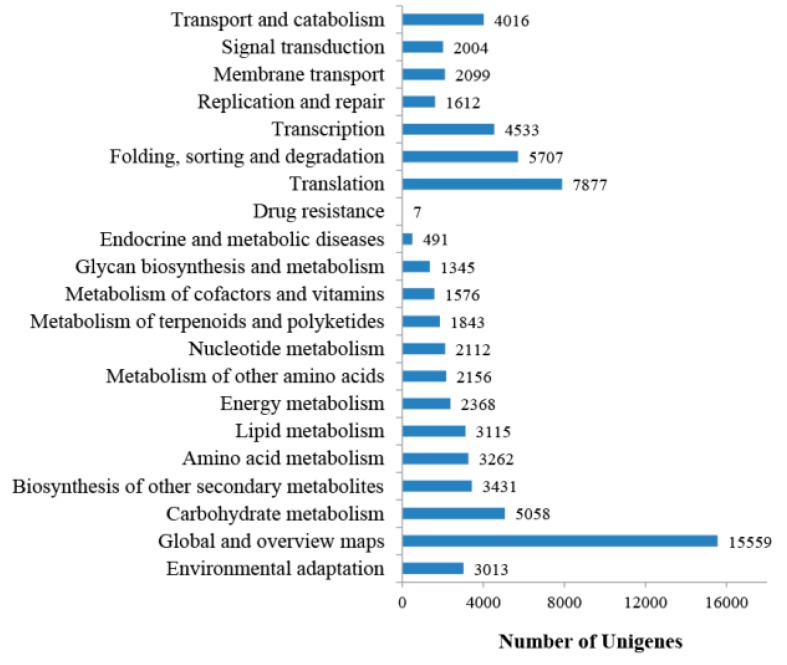
Kyoto Encyclopedia of Genes and Genomes (KEGG) pathway annotation of all unigenes.

Gene Ontology (GO) assignments were used to classify the functions of the putative genes. In our work, a total of 9284 unigenes were assigned to three GO ontologies that were further classified into 44 categories. As shown in Figure S2, the number of unigenes assigned to the molecular function ranked the highest (7144, 76.95%), followed by biological process (6159, 66.34%) and cellular component (5173, 55.72%). Under the category of biological process, “cellular process” and “metabolic process” were the most abundant. “Cell” and “cell part” were overrepresented GO terms of the cellular component. “Catalytic activity” and “binding” covered the majority of the categories of molecular functions. In addition, a very small number of unigenes in GO terms of behavior (one unigene), biological adhesion (two unigenes), extracellular matrix part (three unigenes), and metallochaperone activity (three unigenes) were found in this study (Figure S2). The Kyoto Encyclopedia of Genes and Genomes (KEGG) database is a knowledge base for systematically analyzing the gene products of metabolic processes. A total of 58,636 unigenes were assigned to 132 KEGG pathways and grouped into six KEGG biochemical pathways: genetic information processing, organismal systems, cellular processes, environmental information processing, human diseases, and metabolism (Figure 1).

### 2.2. Overview of Small RNA Sequencing and Annotation

**Figure 2:**
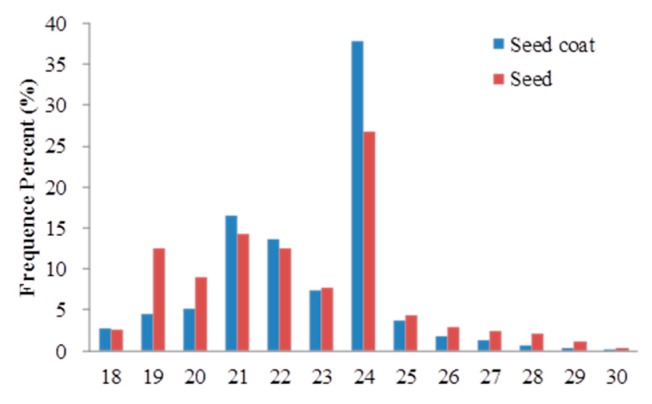
Length distribution and abundance of sRNAs in *P. polyphylla* var. *yunnanensis*.

In this study, four libraries from two replicates of seeds and seed coats were independently sequenced. More than 12.5 M raw reads of each replicate were obtained (Table S2). After filtering out low-quality reads, contaminations, adaptors, and those reads smaller than 18 nt, the average clean reads were 12,444,074 and 12,431,325 for seed coats and seeds, respectively (Table 2). The lengths of these sRNAs were distributed in 18–30 nt, but the reads of 21–24 nt were the most abundant (Figure 2). In particular, the sequences of 24 nt in length were most abundant in the seed coat and seed libraries, accounting for 37.82% and 27.83% of the total sequences, respectively. This was consistent with the typical miRNA in size. Meanwhile, the result indicated that some other RNA types were contained in these reads. Subsequently, we annotated all the reads of four libraries using the Blast program to search against Genebank (http://www.ncbi.nlm.nih.gov/) and Rfam (http://rfam.sanger.ac.uk). All non-coding small RNAs, including snRNAs, snoRNAs, tRNAs, rRNAs, and miRNAs were annotated in each library; their numbers are summarized in Table S3. A total of 310,500, 209,715, 226,502, and 134,051 reads were annotated as miRNA sequences in two replicate libraries of seeds and seed coats, respectively (Table S3). These data suggest that known miRNAs are only a small portion of these complicated ingredients in sequenced data.

### 2.3. Conserved miRNAs in P. polyphylla var. yunnanensis

To identify conserved miRNAs of *P. polyphylla* var. *yunnanensis*, all miRNA reads resulting from the four small RNA libraries were searched against the miRBase database (release 21) [22]. A total of 310,500, 209,715, 226,502, and 134,051 reads in four libraries of seeds and seed coat, respectively, were homologous with known miRNAs and accounted for 2.69%, 1.57%, 1.83%, and 1.07% of each library, respectively (See Table S2). After merging the four libraries, a total of 263 conserved miRNAs were detected, in which 116 conserved miRNAs were assigned 83 families and another 147 conserved miRNAs were not classified into any families (Table 2 and Table S4). As shown in Table S4, the most abundant are miR166 (six members), miR159 (five members), and miR156/157 (five members). Of 263 miRNAs, 104 were expressed in seeds and seed coats, which accounted for 39.24%; 44 were not detected in the seed coat library and 115 were not detected in the seed library (Table S4). These results indicated that the expression level of miRNAs was significantly different among tissues and many miRNA families displayed a tissue-specific expression pattern.

High-throughput sequencing produced the different frequency of miRNA sequences, which can be used as an index for determining the relative abundance of miRNAs in *P. polyphylla* var. *yunnanensis*. The different miRNA families exhibited variable frequencies in abundance. Take commonly expressed miRNAs in seeds and seed coats, for example: miR6150, miR9484, miR166k, miR396f, and miR156a were the top five miRNAs in terms of abundance in seeds, whereas miR159a, miR894, miR396f, miR6150, and miR9484 had the highest abundance in seed coats (Table S4). We found that miR396f exhibited the largest difference in expression abundance between seeds and seed coats, followed by miR6150, miR5577, and miR159a (Table S4). In addition, some members of the miRNA families were prone to be expressed in specific tissue, e.g., miR166a-3p were found to have a low expression in seed coats and a high expression in seeds, but miR166h-3p had a high expression in seed coats and a low expression in seeds (Table S4).

### 2.4. Novel miRNAs in P. polyphylla var. yunnanensis

In our study, the unannotated small RNAs (i.e. 18–30 nt sRNAs in length) were searched against transcript sequences using Mireap software (http://sourceforge.net/projects/mireap/) with several criteria to identify the novel miRNAs and obtain their corresponding precursor sequences (see Section 4). A total of 768 novel miRNAs in 1174 transcripts were identified after merging the four small libraries (Table S5). miRNA* sequence can be used as a criterion for the identification of novel miRNAs. In this study, 679 miRNA* sequences were found in 541 precursors of novel miRNAs, of which 144 precursors contained both miRNA and miRNA* sequences (Table S5). The length of the mature sequences of novel miRNAs ranged from 20 to 24 nt for the mature sequences, in which the majority was 21 nt long. The precursor sequences of these novel miRNAs had an average length of 157 nt and the minimum free energy (MFE) of their second structures varied from −225 kcal/mol to −18.04 kcal/mol, with an average of −57 kcal/mol (Table S5). In addition, we found that the same mature miRNAs originated from the different transcripts. For example, novel-miR409 was generated from two unigenes and novel-miR108 was generated from 11 unigenes (Table S5). The lower abundance of all the novel miRNAs was shown when compared with conserved miRNAs (Tables S4 and S5). The novel miRNAs showed different expression levels, and their reads ranged from 5 to 54,910. As shown in Table S5, novel-miR409 and novel-miR234 were the most abundant miRNAs. Most novel miRNAs had variable reads, ranging in number from 5–10 in abundance, accounting for 54.47% (Table S5). In addition, we found that a small number of novel miRNAs were detected in seeds and seed coats and most of them (85.12%) were expressed in a tissue-specific manner (Table S5), in which 421 novel miRNAs were observed in seed coats, and 231 in seeds, respectively.

### 2.5. Target Prediction of Novel and Conserved miRNAs

Plant miRNAs play important roles in various biological and metabolic processes by cleaving their target mRNAs or suppressing their translation. To understand the biological functions of all identified miRNAs, the target genes of 263 conserved miRNAs and 768 novel miRNAs were predicted using online psRNATarget sever with the default setting (http://plantgrn.noble.org/psRNATarget/) [3]. A total of 14,711 target transcripts were identified, in which 610 were targeted by both conserved miRNAs and novel miRNAs, 3087 were specifically targeted by conserved miRNAs, and 11,014 by novel miRNAs (Table S6). Target gene prediction in this study showed that miRNAs can also target transcription factors (TFs), including squamosa promoter binding protein-like (SPL), APETALA2 (AP2), myeloblastosis-related proteins (MYB), and WRKY proteins (Table S6). Of them, MYB-related TFs and ARF TFs were the most abundant in number (49 for MYB and 37 for ARF TF) (Table S6).

GO assignments were used to classify the functions of the putative target genes. A total of 1296 unigenes (8.81%) were assigned 40 functional groups and allocated to three specific GO categories (Figure S3A). We found that these targets mainly focused on the most major functions of three GO term categories, just like all unigenes (Figures S2 and S3A). For example, the targets still showed as the most abundant in “cellular process” and “metabolic process” of the biological processes category, although they had a smaller number than all the unigenes (Figures S2 and S3A). When considered separately, the targets for conserved miRNAs and novel miRNAs were enriched in each GO term; the targets of novel miRNAs had a similar number to those of conserved miRNAs in most cases (Figure S3B). However, we found that some targets were only enriched in the specific GO terms. For example, five enriched GO terms “translation regulator”, “locomotion”, “rhythmic process”, “death”, and “viral reproduction” were enriched in the targets of novel miRNAs, whereas “extracellular region part” and “molecular transducer” were only found to be enriched in the targets of conserved miRNAs (Figure S3B). These results revealed that the novel and conserved miRNAs might have been involved in the specific processes and conduct the specific functions.

**Figure 3:**
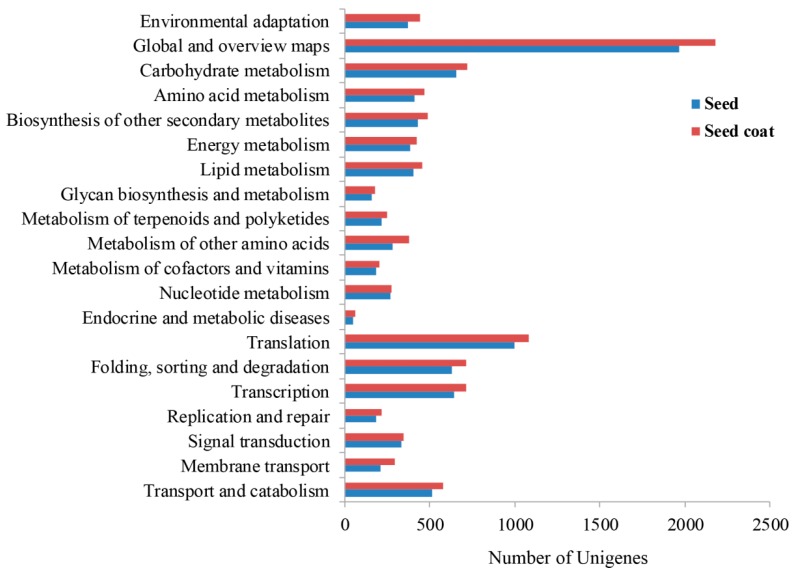
KEGG pathway annotation of the targets of seeds and seed coats.

Pathway enrichment analysis was performed to further connect the biological functions of these targets. In this study, a total of 5217 target genes were significantly matched in this database, and assigned to 20 KEGG pathways (Figure S4). Our results showed that these targets involved in cellular processes were mainly related to the pathway of transport and catabolism (Figure S4). In the metabolism pathway, we found that most of the targets participated in the secondary metabolites and primary metabolites (carbohydrate metabolism and amino acid metabolism) (Figure S4). Previous study indicated that organic acid, alkaloid, and phenolic compounds are the main germination inhibitors [15]. Further analysis indicated that some targets were the putative genes that were involved in the alkaloid pathway or flavonoid biosynthesis pathways (Table S6). These results suggested that miRNAs play an important role in directly regulating the biosynthesis of these germination inhibitors. In addition, the transcription factor families participate in the biosynthesis of many secondary metabolites. For example, flavonoid pathway genes are known to be coordinately induced by a common set of proteins, comprised of MYB, basic helix-loop-helix (bHLH) proteins plus WD40 repeat proteins [23]. In our results, the members of MYB and bHLH transcription factors were targeted by different miRNA families (Table S6). Therefore, these results demonstrated that miRNAs also indirectly regulate the biosynthesis of second metabolites by targeting these transcription factors. In addition, 2056 (39.41%) unigenes were found to be related to genetic information processing, such as “Translation”, “Folding, sorting and degradation”, “Replication and repair”, and “Transcription” (Figure S4). Overall, these targets were mainly involved in the cell, metabolism and genetic information processing during the maturity of seeds in *P. polyphylla* var. *yunnanensis*. When we separately compared KEGG classifications of the target genes between seed miRNAs and seed coat miRNAs, we found that “Global and overview maps”, including “metabolic pathways” and “biosynthesis of secondary metabolites”, had large differences in the KEGG classifications (Figure 3). 

## 3. Discussion

*P. polyphylla* var. *yunnanensis* is a traditional Chinese medicine and has hemostasis, antimicrobial, and anti-inflammatory activity due to the existence of steroid saponins. However, the long dormancy period of seeds limits the artificial cultivation at a large scale. With the extinction of wild plant resources, study of the molecular mechanisms of seed dormancy is important to guide the breaking of seed dormancy in *P. polyphylla* var. *yunnanensis*. miRNAs, as important regulatory factors, play an important role in seed dormancy, but are not studied in *P. polyphylla* var. *yunnanensis* at present. High-throughput sequencing and bioinformatic approaches are efficient means for identifying miRNAs at the genome-wide scale. However, as a non-model plant with large genome size, *P. polyphylla* var. *yunnanensis* is rarely studied at the molecular level except for its medicinal value. To identify these miRNAs, we thus sequenced the transcriptome data as a reference genome of *P. polyphylla* var. *yunnanensis*. In this study, a total of 146,671 unigenes were identified and outnumbered by approximately 3-fold the unigenes found in the previous study [21]. One possible reason was that the different fragments from less expressed genes or many allelic variants were identified at a greater depth of sequencing. The general function of miRNAs is to regulate gene expression by mediating the cleavage of target mRNAs or repressing their translation. The acting site of miRNA is supposed to locate the complementary sequence of mRNA. Due to the limited genome information on *P. polyphylla* var. *yunnanensis*, the more identified unigenes will be favor of the finding of miRNAs and their targets in this species.

This is the first study to report the identification of a large number of miRNAs in *P. polyphylla* var. *yunnanensis*. Among four small RNA libraries of seeds and seed coats for this species, sequences with 24 nt in length dominated the libraries (Figure 2). This result was consistent with reports in maize and rice, but not in *Populous trichocarpa*, where 21 nt small RNAs were the most abundant [24,25,26]. These results suggested that the small RNA transcriptomes might be complex and significantly different across plant species, in particular the phylogenetically distant plant species. Our results indicated that the length of the majority of miRNAs ranged from 20 to 24 nt, which is typical of Dicer-processed products. In *Panax notoginseng*, 41 conserved miRNA families were identified [27], whereas our results indicated that at least 83 conserved miRNA families were detected because many conserved miRNAs in this species belong to undefined families (Tables S4 and S5). As in a previous study, the conserved miR156, miR164, miR159, and miR396 also targeted SPL, NACs, MYB, and GRF TFs in this study [6,28]. In lettuce, miR156 and miR172 can regulate the *delay of germination1* (*DOG*1) gene in regulating seed dormancy in response to temperature [8]. In *P. polyphylla* var. *yunnanensis*, only several miR156 members were identified (Table S4). In addition, the miR172 family is widely found in angiosperms and always targets Apetala2 (AP2) transcription factor. Although no miR172 members were found in *P. polyphylla* var. *yunnanensis*, we found that AP2 genes were regulated by other conserved miRNA families, such as miR393a, miR1162, miR5648, miR7698, and some novel miRNAs (Table S6). Based on these results, we inferred that a different dormant mechanism might exist between *P. polyphylla* var. *yunnanensis* and lettuce owing to the different type of seed dormancy. Lettuce belongs to a dormant type of thermoinhibition that results in reduced germination during warm seasons [29,30], whereas *P. polyphylla* var. *yunnanensis* employs a type of morphophysiological dormancy [11].

Our results also indicated that the unigenes of the seed coat apparently outnumbered those of the seed, as well as miRNAs and their targets. As the normal mature tissue, the seed coats might have a more complex gene regulatory system than dormant seeds do in *P. polyphylla* var. *yunnanensis*. Based on the GO and KEGG functional classifications, all unigenes were mainly assigned into cell and metabolic process (Figure 1 and Figure S2). When we only considered the targets of miRNAs, cell and metabolic process were still the marked functional categories (Figure 3 and Figure S3), suggesting that miRNAs play an important role in these main processes. In addition, it is worth pointing out that many target genes were assigned into the group of metabolic products, in particular secondary metabolites. A previous study indicated that organic acid, alkaloid, and phenolic compounds are the main germination inhibitors [15]. In this study, we found that miRNAs might regulate the biosynthesis of these secondary metabolites by direct and indirect means in seeds and their seed coats (Table S6). Therefore, the inhibitory effects of miRNAs on the seed germination might be via a complex gene regulation network. Accumulating evidence has proven that miR396 family members can regulate key aspects of plant development, hormone signaling, and stress responses by targeting growth-regulating factors (GRFs) [31]. In this study, we found that miR396f exhibited the largest difference in expression abundance between seeds and seed coats. What is the function of miR396f in *P. polyphylla* var. *yunnanensis*? Does it play an important role in differentiation during the maturation of the seed and seed coat? These questions will be worth addressing in a future study.

## 4. Materials and Methods

### 4.1. Plant Materials

The mature dormant seeds and their mesophyll seeds coats of *P. polyphylla* var. *yunnanensis* were collected in Ludian town, Yunnan Province, China. All materials were snap-frozen in liquid nitrogen and then stored at −80 °C. Three biological replicates were used for each sample in transcriptome sequencing and two biological replicates were used for small RNA sequencing. Moreover, each replicate material was collected from an independent individual of *P. polyphylla* var. *yunnanensis*.

### 4.2. RNA Preparation, Library Construction, and Illumina Sequencing

Total RNA was isolated from each material using RNeasy Plant Mini Kit (Qiagen, Valencia, CA, USA), and then treated with DNase I kit (TakaRa, Kyoto, Japan). Before RNA sequencing, the quality and quantity of total RNA were examined using an Agilent 2100 Bioanalyzer (Agilent, Santa Clara, CA, USA). The mRNA sequencing was constructed using previously described methods [32]. Briefly, total RNA were extracted and subsequently prepared for mRNA-Seq on the Illumina Genome Analyzer II following the manufacturer’s instructions (Illumina Inc., San Diego, CA, USA) and cDNA libraries were constructed using random hexamer primers and samples were sequenced using an Illumina HiSeq™ 2000 platform (Illumina Inc.).

In addition, the total RNA extraction was constructed as in the above-described method for small RNA library construction and Illumina sequencing. Firstly, 18–30 nt sRNAs were purified from a 15% denaturing polyacrylamide gel and then ligated with 5′ and 3′ adaptors. After being reverse-transcribed, sRNAs were amplified by PCR, and then deep sequenced on an Illumina HiSeq 2000 platform (Illumina Inc.). In this study, three cDNA libraries and two small RNA libraries for seeds and seeds were constructed and sequenced, respectively.

### 4.3. Transcript Assembly and Annotation

After filtering the mRNA sequence raw data (adaptor, low-quality with more than 20% Q <10 bases, PCR duplication and reads containing unknown base more than 5%), de novo assembly into longer and gapless contigs was performed by using the Inchworm module in the Trinity tool (the Broad Institute, Boston, MA, USA) [33]. Then the contigs were clustered with the Chrysalis module by constructing a de Bruijn graph for each cluster and partitioning the reads among the disjoint graphs. Subsequently, the Butterfly module was used to trace reads through the path. Finally, each unigene sequence from each sample’s assembly was combined into a unique all-unigene with clustering software: TGICL version 2.0.6 [34].

To annotate the assembled transcripts, the BLAST program was used to search against non-redundant (Nr) and KEGG (*E*-value < 0.00001). Functional classification of target genes was achieved through Web Gene Ontology Annotation Plot (WEGO, http://www.geneontology.org/) [35]. Open reading frames (ORFs) of the individual assembled transcripts were predicted using the “getorf” program of EMBOSS package [36], by which the longest ORF was extracted for each transcript. All transcripts were searched against the PlntfDB database (http://plntfdb.bio.uni-potsdam.de/) using HMMER v3.0 (*E*-value < 0.0001) to screen the transcription factors from transcriptomes. The RNA-Seq raw data of this study have been deposited in NCBI Sequence Read Archive (SRA) with accession number: SRR5003758, SRR5003774, SRR5003775, SRR5003776, SRR5003777 and SRR5003778.

### 4.4. Bioinformatics Analysis of sRNAs

After removal of adaptor sequences and filtering out low-quality reads, the cleaned sRNAs reads were mapped to the GenBank (http://www.ncbi.nlm.nih.gov/) and Rfam (version 11.0, http://rfam.xfam.org/) databases to discard rRNA-, scRNA-, snoRNA-, snRNA-, and tRNA-associated reads. The conserved miRNAs were identified and annotated by BLASTn search against miRBase (release 21, http://www.mirbase.org/) without any mismatches. The novel mature miRNAs were identified using the Mireap program (version 0.1, http://sourceforge.net/projects/mireap/). Then their precursor sequences were obtained by perfect mapping into transcript sequence data to predict the hairpin-like secondary structure. The parameters used to identify novel miRNAs were as follows: minimal miRNA sequence length is set to 18 nt; maximal miRNA sequence length is set to 25 nt; minimal miRNA reference sequence length is set to 20 nt; maximal miRNA reference sequence length is set to 23 nt; maximal copy number of miRNAs on reference is set to 20 nt; maximal free energy allowed for a miRNA precursor is set to –18 kcal/mol; maximal space between miRNA and miRNA* is set to 300 nt; maximal bulge of miRNA and miRNA* is set to 16 nt; minimum bulge of miRNA and miRNA* is set to 4 nt; maximal asymmetry of miRNA/miRNA* is set to 4 nt; and flank sequence length of miRNA precursor is set to 20 nt. Target genes of both conserved and novel miRNAs were predicted by using the online psRNATarget server with the default settings (http://bioinfo3.noble.org.psRNATarget/) [3].

## 5. Conclusions

In this study, we first identified 263 conserved miRNAs and 768 novel miRNAs in seeds and seed coats of *P. polyphylla* var. *yunnanensis* by high-throughput sequencing technologies when combined with the transcriptome data as the genome reference. Meanwhile, the targets of the conserved and novel miRNAs were predicted and functionally annotated, suggesting s that these miRNAs were mainly involved in the cell, metabolism and genetic information processing by direct and indirect regulation patterns in dormant seeds of *P. polyphylla* var. *yunnanensis*. Such study provides a basis to further explore the molecular mechanism of seed dormancy in this species.

## Figures and Tables

**Table 1:** Summary information of sequenced and assembled transcriptome data in seed and seed coats.

Sequences	Samples	
	Seed Coat	Seed
Reads		
Total Raw Reads (MB)	63.33	63.33
Total Clean Reads (MB)	59.42	59.44
Total Clean Reads (MB)	59.42	59.44
Total Clean Bases (GB)	8.91	8.92
Clean Reads Q 20 (%)	97.21	97.42
Unigenes		
Number	135,008	90,419
Mean Length	955	1241
Total unigenes	14,6671	

**Table 2:** Summary of high-throughput sequencing and assembling results of *P. polyphylla* var. *yunnanensis* small RNAs.

Reads	Samples		
	Seed Coat	Seed	Total
Raw reads	13,045,488	12,651,991	
High quality	13,006,850	12,606,706	
3′ Adaptor null	45,875	42,137	
Insert null	2433	608	
5′ Adaptor contaminants	13,837	6134	
Smaller than 18 nt	500,459	126,650	
Clean reads	12,444,074	12,431,325	
Conserved miRNAs	219	148	263
Novel miRNAs	902	640	768

## Supplementary Materials

Supplementary materials can be found at www.mdpi.com/1422-0067/18/1/219/s1.
