# Supplementary material for: Transcriptome-Wide Identification and Prediction of miRNAs and Their Targets in Paris polyphylla var. yunnanensis by High-Throughput Sequencing Analysis
Source: Int J Mol Sci. 2017 Jan 22;18(1):219. doi: 10.3390/ijms18010219 (PMC5297848; doi:10.3390/ijms18010219)
Supplement: Supplementary file 1 [file ijms-18-00219-s001.zip › ijms-170045-final-supply.pdf]

Supplementary Materials: Transcriptome-Wide Identification and Prediction of miRNAs and Their Targets in *Paris polyphylla* var. *yunnanensis* by High-Throughput Sequencing Analysis

Li-Zhen Ling, Shu-Dong Zhang, Fan Zhao, Jin-Long Yang, Wen-Hui Song, Shen-Min Guan, Xin-Shu Li, Zhuang-Jia Huang, and Le Cheng

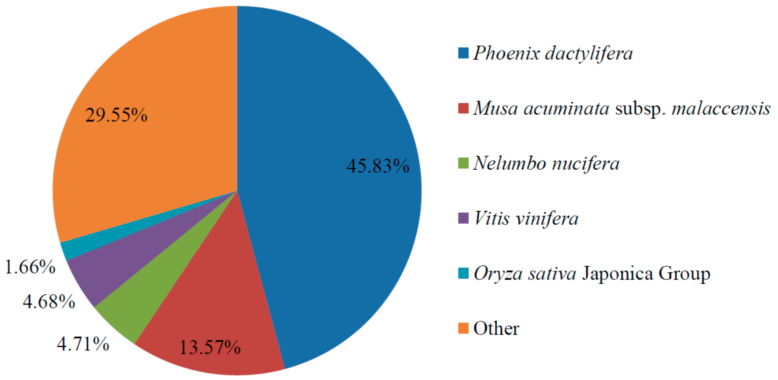

Figure S1. Species distribution of unigenes matching the top five species using Blastx in the Nr database.

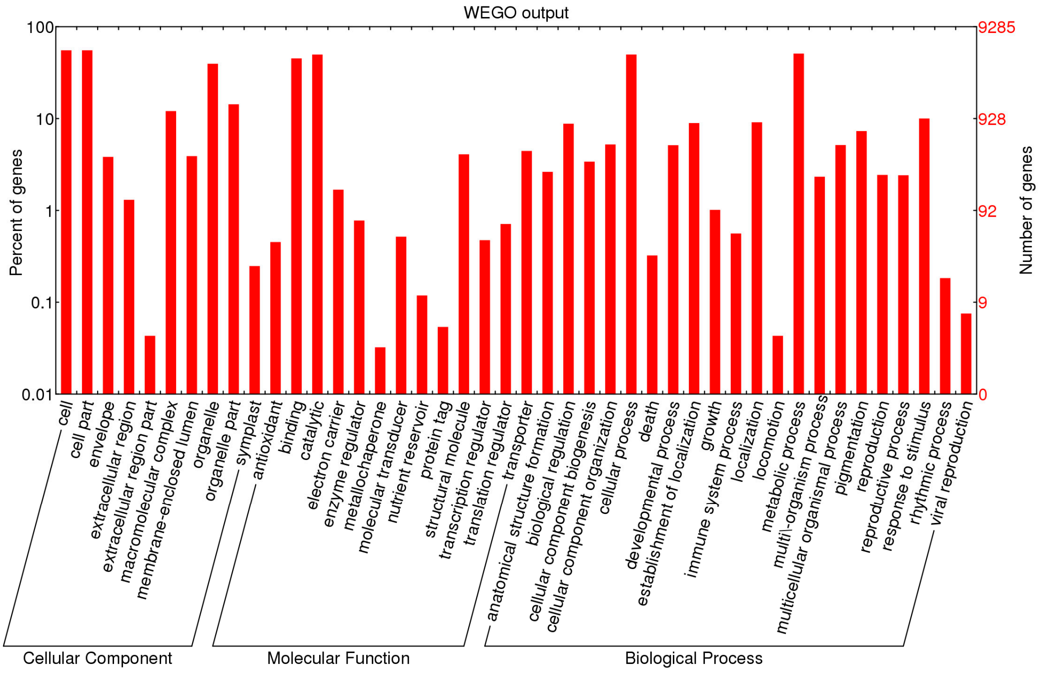

Figure S2. Gene Ontology (GO) categories assigned to all unigenes.

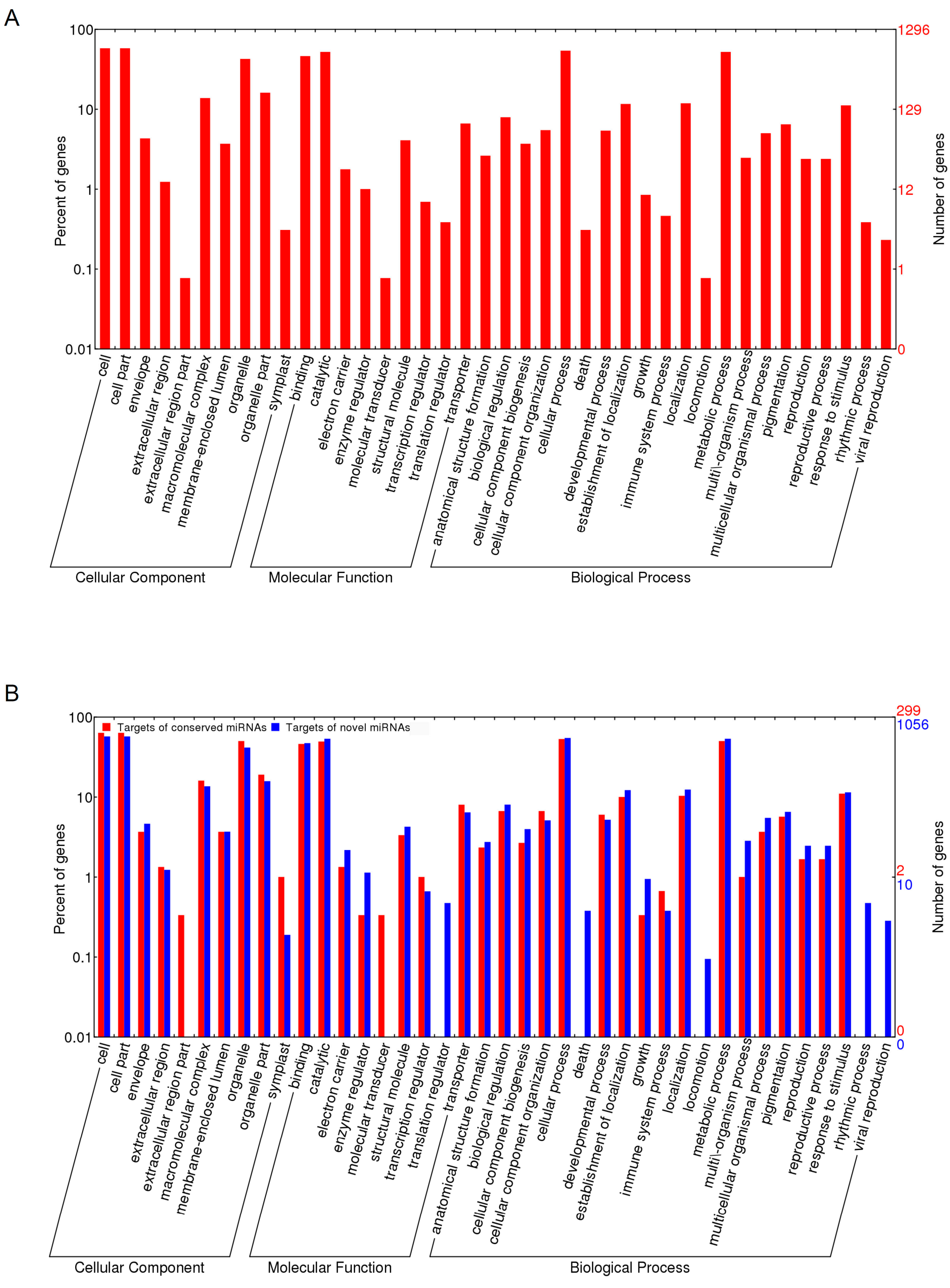

Figure S3. GO categories assigned to all targets (**A**); and the targets of conserved and novel miRNAs (**B**).

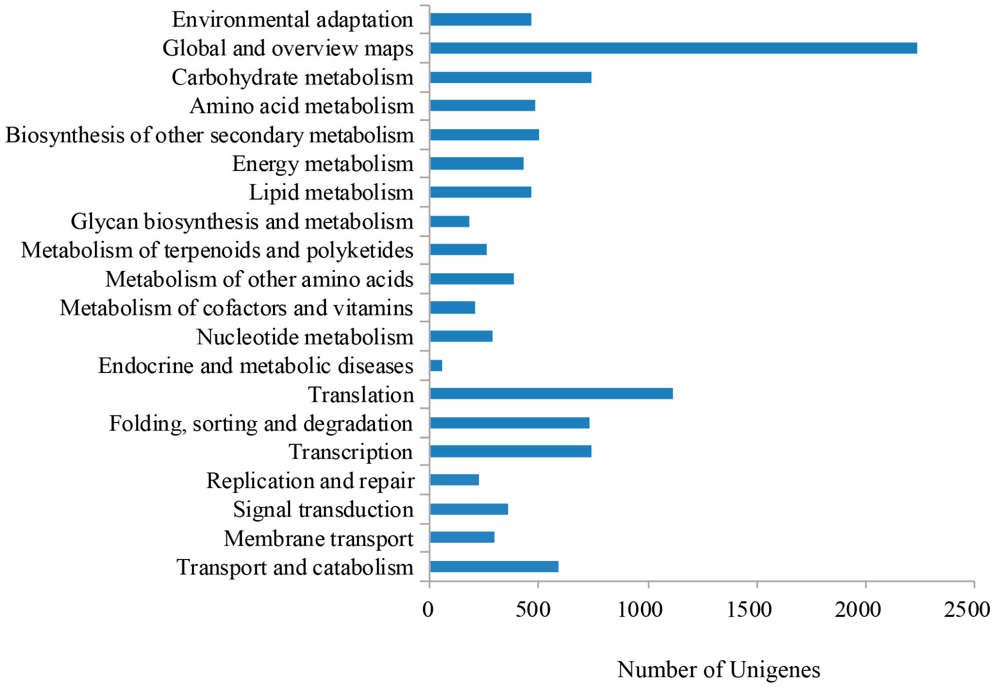

Figure S4. Kyoto Encyclopedia of Genes and Genomes (KEGG) pathway annotation of all targets.

Table S1. Detailed information on each sequenced and assembled transcriptome library in seed and seed coats.

Sequences	Samples					
	**Seed Coat-1**	**Seed Coat-2**	**Seed Coat-3**	**Seed-1**	**Seed-2**	**Seed-3**
Reads						
Total Raw Reads (MB)	63.33	63.33	63.33	63.33	63.33	63.33
Total Clean Reads (MB)	59.61	58.93	59.72	59.09	58.98	60.26
Total Clean Bases (GB)	8.94	8.84	8.96	8.86	8.85	9.04
Clean Reads Q20 (%)	97.4	97.15	97.08	97.54	97.2	97.54
Unigenes						
Number	83,715	79,682	87,539	38,115	39,261	36,138
Mean Length	673	675	655	790	776	770

Table S2. Statistics of high-through sequencing and assembling results of each *P. polyphylla* var. *yunnanensis* small RNA library.

Sequences	Samples			
	**Seed Coat-1**	**Seed Coat-2**	**Seed-1**	**Seed-2**
Raw reads	12,561,372	13,529,604	12,689,441	12,614,541
High quality	12,525,800	13,487,899	12,640,627	12,572,784
3′ Adaptor null	48,263	43,450	46,933	37,341
Insert null	2471	2394	783	432
5′ Adaptor contaminants	17,365	10,309	6771	5497
Smaller than 18 nt	915,201	85,717	207,993	44,506
Clean reads	11,542,355	13,345,793	12,377,969	12,484,680
Conserved miRNAs	140	158	81	134
Novel miRNAs	489	413	299	341

Table S3. Statistics of annotated small RNA data of two replicate libraries in seed and seed coat of *P. polyphylla* var. *yunnanensis*.

Category	Unique sRNAs %			
	Seed Coat-1	Seed Coat-2	Seed-1	Seed-1
rRNA	0	0.09	12.76	8.08
snoRNA	0	0	0.04	0.07
tRNA	0	0.02	3.21	5.10
snRNA	0	0.01	0.22	0.17
miRNA	2.69	1.57	1.83	1.07
unannotated	97.31	98.31	81.94	85.50
